# HER2 confers drug resistance of human breast cancer cells through activation of NRF2 by direct interaction

**DOI:** 10.1038/srep07201

**Published:** 2014-12-03

**Authors:** Hyo Jin Kang, Yong Weon Yi, Young Bin Hong, Hee Jeong Kim, Young-Joo Jang, Yeon-Sun Seong, Insoo Bae

**Affiliations:** 1 Department of Oncology, Lombardi Comprehensive Cancer Center, Georgetown University, Washington DC, 20057, USA; 2 Department of Radiation Medicine, Lombardi Comprehensive Cancer Center, Georgetown University, Washington DC, 20057, USA; 3 Department of Nanobiomedical Science and BK21 PLUS Research Center for Regenerative Medicine, Dankook University, Cheonan, Korea

## Abstract

Overexpression and/or activation of HER2 confers resistance of cancer cells to chemotherapeutic drugs. NRF2 also gives drug resistance of cancer cells through induction of detoxification and/or drug efflux proteins. Although several upstream effectors of NRF2 overlapped with the downstream molecules of HER2 pathway, no direct link between HER2 and NRF2 has ever been established. Here, we identified that co-expression of a constitutively active HER2 (HER2CA) and NRF2 increased the levels of NRF2 target proteins, HO-1 and MRP5. We also identified HER2CA activated the DNA-binding of NRF2 and the antioxidant response element (ARE)-mediated transcription in an NRF2-dependent manner. In addition, NRF2 and HER2CA cooperatively up-regulated the mRNA expression of various drug-resistant and detoxifying enzymes including GSTA2, GSTP1, CYP3A4, HO-1, MRP1, and MRP5. We also demonstrated that NRF2 binds to HER2 not only in transiently transfected HEK293T cells but also in HER2-amplified breast cancer cells. Functionally, overexpression of HER2CA gave resistance of MCF7 breast cancer cells to either paraquat or doxorubicin. Overexpression of dominant negative NRF2 (DN-NRF2) reduced the HER2CA-induced resistance of MCF7 cells to these agents. Taken together, these results suggest that active HER2 binds and regulates the NRF2-dependent transcriptional activation and induces drug resistance of cancer cells.

Human epidermal growth factor receptor 2 (HER2) is a member of epidermal growth factor receptor (EGFR)/HER2 family receptor tyrosine kinases (RTKs)^1^,^2^. As an oncogene, HER2 is amplified in approximately 25–30% of human breast and ovarian cancers^1^,^2^. In addition, somatic mutations in the HER2 kinase domain have been identified in a small subset of lung cancers^1^. Overexpression/overactivation of HER2 triggers deregulated signal transduction cascades that induce tumorigenesis of human cells and maintain tumor cells growth and/or survival^1^. HER2 also induces drug resistance of tumor cells including breast cancers. As an example, HER2 induces taxane resistance in breast cancers through signal amplification that: 1) induces drug efflux pumps including the ATP-binding cassette, sub-family B, member 1 (ABCB1) and ABCC3; 2) enhances the expression of drug metabolism proteins such as glutathione S-transferase P1 (GSTP1) and cytochrome P450 3A4 (CYP3A4); and 3) stimulates the expressions of cell survival proteins such as survivin, p21, and p53, etc^3^. Increased signaling by HER2 also up-regulates phosphorylation of estrogen receptor α (ERα) that causes resistance of breast cancer to endocrine therapy^4^. Recently, it has been reported that HER2 also up-regulates the expression of the breast cancer resistance protein (BCRP)^5^ through interaction with EGFR to render resistance to aromatase inhibitors^6^. In these cases the HER2-mediated signal transduction, such as the PI3K/AKT pathway, is suggested as the basis of HER2-mediated drug resistance^7^.

Nuclear factor erythroid 2-related factor 2 (NRF2) is a master transcriptional regulator that activates various genes which are involved in oxidative stress response, detoxification, and drug resistance^8^,^9^,^10^,^11^. NRF2 binds to the antioxidant response element (ARE) in the promoter regions of its target genes to activate their transcription^12^. The level of NRF2 is tightly regulated by the Kelch-like erythroid cell-derived protein with CNC homology-associated protein 1 (KEAP1) through ubiquitin-dependent proteolysis under normal reducing conditions^8^,^9^,^10^,^11^. Stability of NRF2 is also regulated by the glycogen synthase kinase 3β (GSK3β)/β-transducin repeat-containing protein (β-TrCP) axis^13^,^14^,^15^. Additionally, the CR6-interacting factor 1 (CRIF1) also down-regulates NRF2 by proteasome-mediated degradation under either reducing or oxidative stress condition^16^. Recently, it has been reported that NRF2 stability is regulated, through protein-protein interaction to compete with or sterically inhibit KEAP1, by various proteins including p21^17^, the Wilms tumor gene on X chromosome (WTX)^18^, p62^19^, the partner and localizer of BRCA2 (PALB2)^20^, the dipeptidyl peptidase III (DPP3)^21^, and the breast cancer susceptibility gene 1 (BRCA1)^22^. In lung, pancreatic as well as colorectal cancer cells, activation of NRF2 may enhance tumor cell proliferation and/or confers resistance to various chemotherapies^8^,^9^,^10^,^11^,^23^,^24^,^25^,^26^,^27^,^28^,^29^. Although stability of NRF2 can be regulated by diverse post-translational modifications such as phosphorylations by various upstream kinases^10^, currently there is no direct link between NRF2 and HER2 in the regulation of drug resistance of cancer cells.

In this study, we demonstrated that HER2 activates NRF2 transcriptional activity through direct protein-protein interaction and induces a subset of NRF2-target gene expression in human breast cancer cells.

## Results and Discussion

### Co-expression of NRF2 and active HER2 induces the expression of NRF2-target gene expressions in MCF7 cells

**Figure 1:**
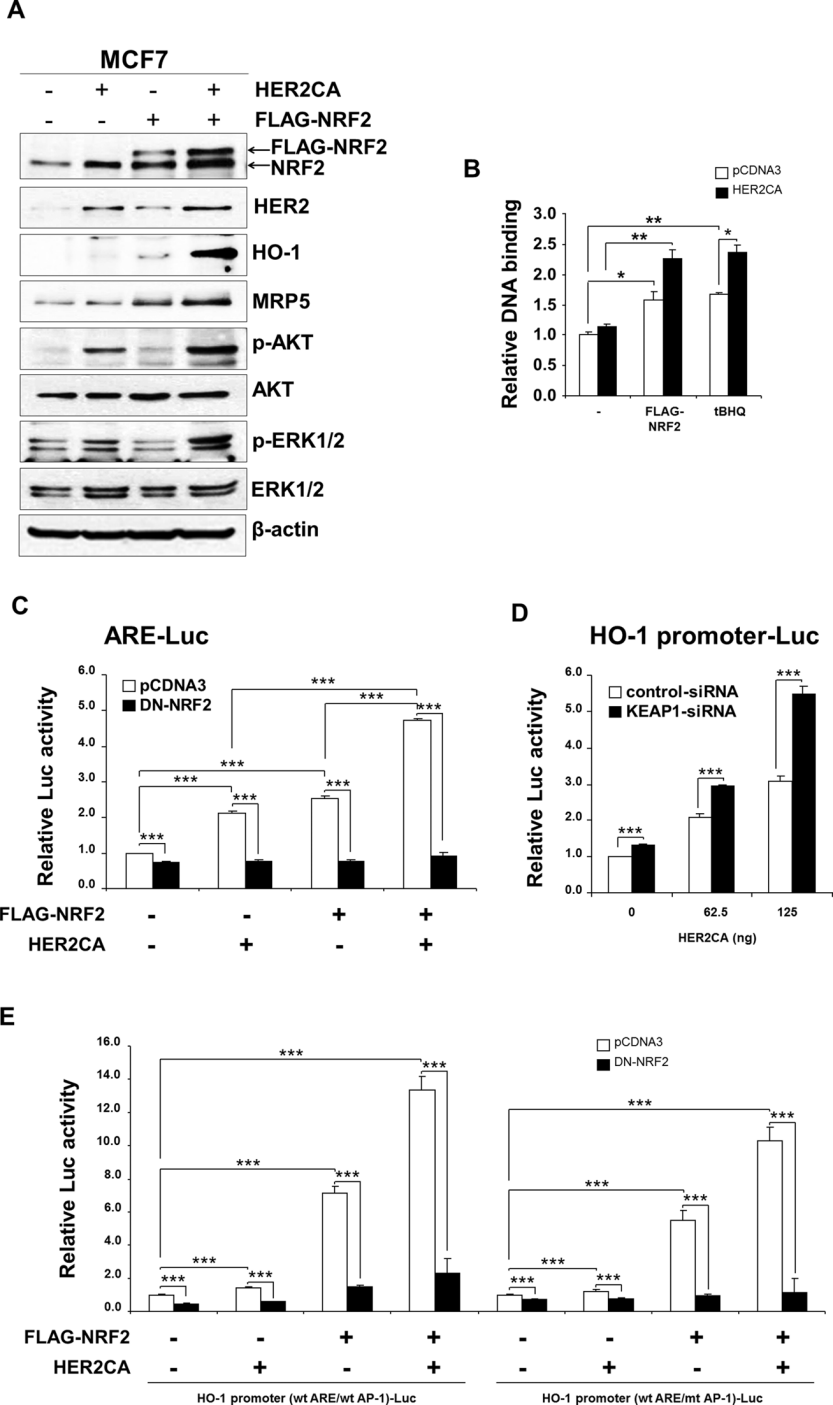
Co-expression of HER2CA and FLAG-NRF2 induces the expressions of NRF2-target proteins and increased the NRF2-dependent transcription in MCF7 cells. (A) Overexpression of HER2CA increases the NRF2-induced expressions of HO-1 and MRP5 in MCF7 cells. MCF7 cells were transiently transfected with expression vectors by Lipofectamine PLUS as indicated and subjected to western blot analysis. β-actin was used as a loading control. The blots used in this experiment was cut into several pieces according to estimated M.W. of proteins of interest and probed indicated antibodies. Different pieces of the same protein blots or same samples with same conditions of electrophoresis and electrotransfer were used and presented. (B) HER2CA increases NRF2 DNA-binding in MCF7 cells. MCF7 cells were transfected with expression vectors as indicated and NRF2 DNA-binding assay was performed. As a control, MCF7 cells were treated with 100 µM of tBHQ for 8 hr. (C) HER2CA increases the ARE-Luc reporter gene activity in an NRF2-depedent manner. MCF7 cells were transfected with expression vectors as indicated with Lipofectamine PLUS and luciferase activity was determined as described in Materials and methods. (D) HER2CA increased transcriptional activation of HO-1 promoter by endogenous NRF2. MCF7 cells were transfected with HO-1 promoter-Luc and increasing amounts of HER2CA expression vector in the absence or presence of KEAP1 knockdown. Luciferase assay were performed as described in (C). (E) HER2CA increases the NRF2-dependent transcription of HO-1 promoter. MCF7 cells transfected with expression vectors by Lipofectamine PLUS as indicated in the presence of HO-1 promoter-Luc containing either wild type or mutant AP-1 site. Luciferase activity was determined as in (B). (B,C) Representative data were presented as mean ± SEM of triplicate experiments. Statistically significance was determined by two-tailed Student's t-test. *P ≤ 0.05; **P ≤ 0.01; and ***P ≤ 0.001.

We performed transient transfection of FLAG-NRF2 in the absence or presence of constitutively active HER2 (HER2CA)^30^ in MCF7 cells, and then the expression of proteins were analyzed by western blot analysis. As expected, overexpression of HER2CA increased the levels of phospho-AKT (S473) and phospho-ERK1/2 (T202/Y204) (Figure 1A). In addition, HER2CA increased both basal and overexpressed NRF2 protein. Overexpression of NRF2 also induced the expressions of NRF2 target proteins such as heme oxygenase-1 (HO-1) and multidrug-resistant protein 5 (MRP5). Interestingly, co-expression of FLAG-NRF2 and HER2CA markedly increased the expression of NRF2-targets, HO-1 and MRP5, in MCF7 cells.

To determine the effect of HER2CA on the NRF2-dependent transcription, we first performed ELISA-based DNA-binding assay: MCF7 cells were transfected with FLAG-NRF2 and HER2CA and the nuclear extracts were subjected to DNA-binding assay. As shown in Figure 1B, overexpression of HER2CA alone marginally but significantly induced the DNA-binding activity of endogenous NRF2. Surprisingly, co-expression of HER2CA with FLAG-NRF2 significantly induced NRF2-DNA binding activity. In addition, HER2CA further induced the tert-butylhydroquinone (tBHQ)-induced DNA-binding activity of endogenous NRF2.

Since HER2CA enhanced the NRF2 DNA-binding, the effect of HER2CA on the transcriptional activity of NRF2 was further determined by reporter gene assay using the ARE-Luc reporter containing the ARE sites from the NAD(P)H dehydrogenase, quinone 1 (NQO1) promoter^16^,^31^. MCF7 cells were transiently transfected with the ARE-Luc reporter in various combinations of expression vectors including HER2CA, FLAG-NRF2, and the trans-activation domain lacking dominant negative NRF2 (DN-NRF2, aa 435–605)^16^,^32^. As expected, luciferase activity was induced by FLAG-NRF2 expression and DN-NRF2 antagonized the basal and FLAG-NRF2-induced transcription of the ARE-Luc (Figure 1C). Overexpression of HER2CA alone also increased the transcription from ARE-Luc similar to FLAG-NRF2. Again, DN-NRF2 antagonized HER2CA-induced ARE-Luc activity (Figure 1C). Consistent with DNA-binding activity, co-expression of FLAG-NRF2 and HER2CA markedly enhanced the luciferase activity from the ARE-Luc and DN-NRF2 abolished NRF2/HER2CA-induced transcription (Figure 1C).

To determine the HER2CA effect on the activity of endogenous NRF2, we performed the luciferase assay in MCF7 cells with KEAP1 knockdown. MCF7 cells, transfected with either control-siRNA or KEAP1-siRNA, were further transfected with HO-1 promoter-Luc reporter gene with increasing amounts of HER2CA expression vector. Without NRF2 activator such as tBHQ, KEAP1 knockdown weakly but significantly induced the HO-1 promoter-Luc activity in MCF7 cells (Figure 1D). Consistent with data in Figure 1C, HER2CA induced the luciferase activity from HO-1 promoter-Luc in MCF7 cells transfected with control-siRNA in a dose-dependent manner. Additionally, HER2CA profoundly increased the luciferase activity in a dose-dependent manner when MCF7 cells were transfected with KEAP1-siRNA (Figure 1D).

Since HO-1 promoter contains an AP-1 binding site, we further performed luciferase reporter gene assay with an HO-1 promoter-Luc construct containing mutant AP-1 site. MCF7 cells were transfected with HO-1 promoter-Luc, containing either wild type or mutant AP-1 site, in various combinations of expression vectors (HER2CA, FLAG-NRF2, and DN-NRF2). FLAG-NRF2 induced the luciferase reporter activity regardless of AP-1 site mutation (Figure 1E). Consistent with data in Figure 1d, HER2CA alone also marginally induced the luciferase activity from both reporter constructs. In addition, HER2CA enhanced the FLAG-NRF2-induced transcriptional activation even in the presence of AP-1 site mutation. Interestingly, DN-NRF2 near completely abolished the FLAG-NRF2/HER2CA-induced transcription from HO-1 promoter-Luc containing mutant AP-1 site, while it could not completely abolish the FLAG-NRF2/HER2CA-mediated transcription from HO-1 promoter-Luc containing wild type AP-1 (Figure 1E). These results implicate that HER2CA partly induced the transcription of HO-1 promoter through AP-1 site under these conditions. Taken together, our data suggest that HER2CA induced the expressions of NRF2-target proteins through activation of NRF2 transcription activity.

### HER2CA and NRF2 cooperatively regulates the mRNA expressions of antioxidant and detoxification genes

**Figure 2:**
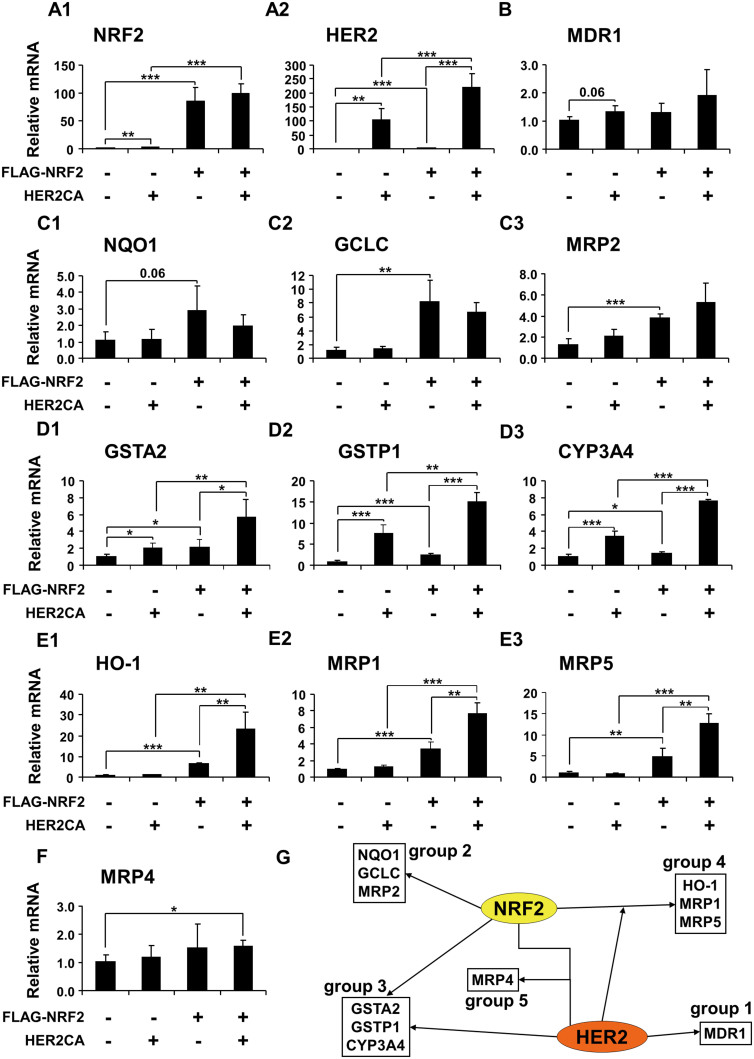
HER2CA and NRF2 regulate the transcription of multiple genes in drug resistance. (A) RT-qPCR analysis of HER2CA and FLAG-NRF2 expression in MCF7 cells after transient transfection of expression vectors by Lipofectamine PLUS as indicated. Transfection was performed as in Figure 1A. (B–F) RT-qPCR analysis of multiple gene expressions. (G) Schematic diagram of the proposed regulation of gene expression by HER2 and NRF2. (A–F) Representative data are shown as mean ± SEM of quadruplicate experiments. Statistically significance was determined by two-tailed Student's t-test. *P ≤ 0.05; **P ≤ 0.01; and ***P ≤ 0.001.

We performed the reverse transcription quantitative polymerase chain reaction (RT-qPCR) analysis to determine the mRNA expressions of NRF2 and HER2 target genes in the presence of exogenously expressed NRF2 and HER2CA. MCF7 cells were transiently transfected with FLAG-NRF2 and HER2CA expression vectors and then RT-qPCR analyses were performed. Overexpression of HER2CA alone in MCF7 cells induced ~2-fold increase of NRF2 mRNA expression (Figure 2A1). However, co-expression of HER2CA and FLAG-NRF2 could not affect the overall mRNA expression of NRF2 compared to FLAG-NRF2 expression alone. Overexpression of FLAG-NRF2 alone also induced ~1.5-fold increase of HER2 mRNA level (Figure 2A2) and co-expression of FLAG-NRF2 and HER2CA further increased the expression of HER2 mRNA. Although specific primers, either for NRF2 or HER2, used in this study could not differentiate the mRNAs from endogenous genes and transfected genes, we cannot rule out a reciprocal regulation of both NRF2 and HER2. Notably, a recent report suggests that HER2 mRNA expression was dependent on the level of NRF2 protein in a HER2-overexpressing ovarian cancer SKOV3 cells^33^.

**Figure 3:**
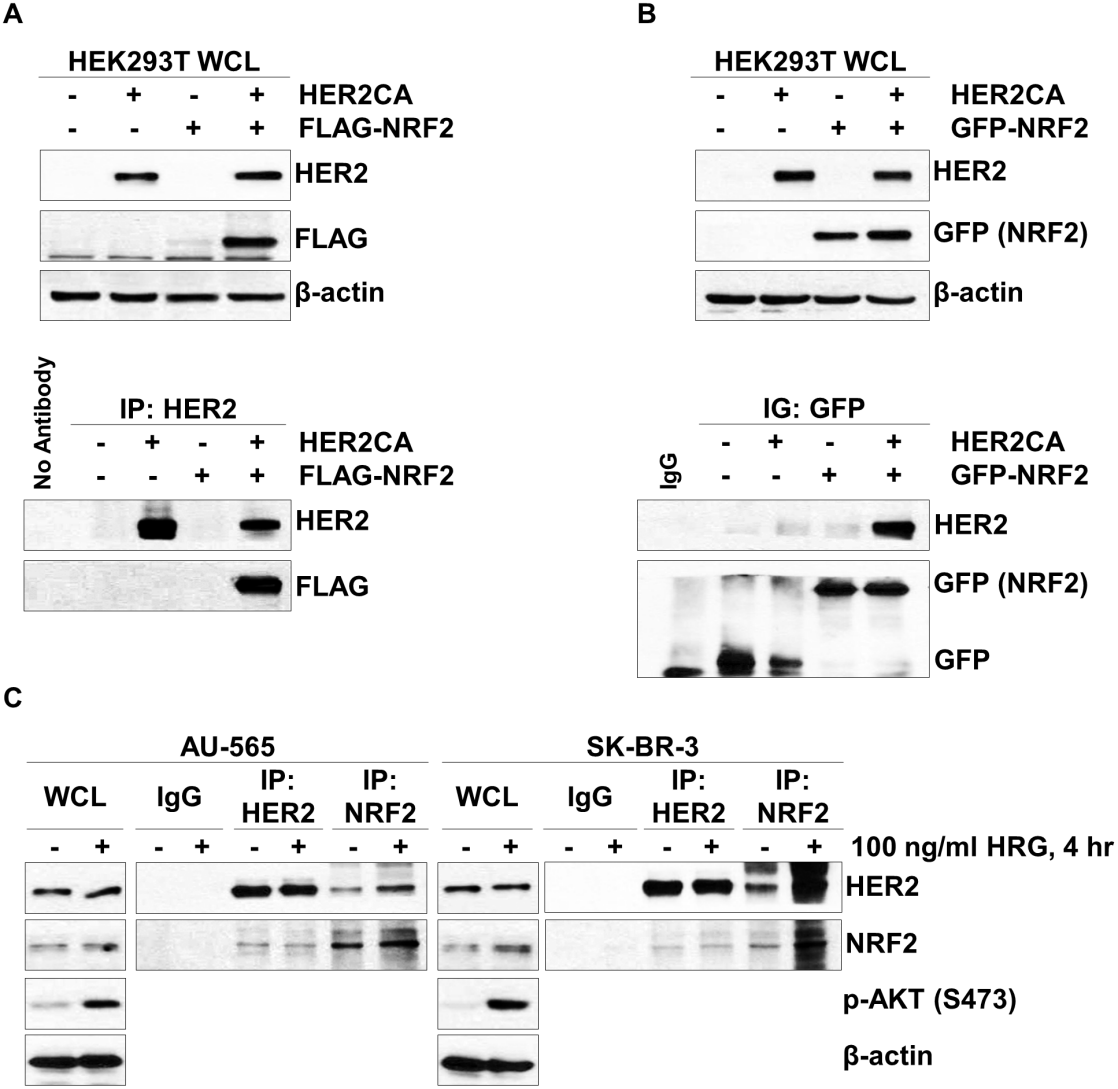
HER2 binds to NRF2. (A,B) HEK293T cells were transfected with HER2CA and (A) FLAG-NRF2 or (B) GFP-NRF2 expression vectors as indicated. (C) HER2 binds to NRF2 in HER2-amplified breast cancer cells. Cells were serum-starved for 24 hr and stimulated by 100 ng/ml of HRG for 4 hr before cell lysis. (A–C) The blots used in this experiment was cut into couples of pieces according to estimated M.W. of proteins of interest and probed with indicated antibodies. Discontinuous lanes from same blots were clearly marked as shown. Immunoprecipitation and western blot analysis were performed with indicated antibodies. β-actin was used as a loading control.

Analysis of RT-qPCR results revealed that at least five groups of genes are differentially regulated by NRF2 and HER2CA: 1) the mRNA expression of multidrug resistance gene 1 (MDR1) in the group 1 was weakly induced by HER2CA under these conditions and less affected by FLAG-NRF2 (Figure 2B); 2) the mRNA expressions of group 2 genes, which are well known NRF2-target genes including NQO1, glutamate-cysteine ligase catalytic subunit (GCLC) and MRP2, were induced by FLAG-NRF2 and less affected by HER2CA (Figure 2C); 3) the mRNA expressions of genes in group 3, such as GSTA2, GSTP1, and CYP3A4, were induced by FLAG-NRF2 and HER2CA, respectively. In addition, mRNA expressions of these genes were synergistically induced by co-expression of FLAG-NRF2 and HER2CA (Figure 2D). Among these, GSTP1 and CYP3A4 was known to be induced by HER2 and contribute to HER2-mediated drug resistance^3^; 4) genes in group 4 (HO-1, MRP1, and MRP5) were also known as NRF2-targets. The mRNA expression of these genes were not induced by HER2CA alone but induced by FLAG-NRF2. However, unlike genes in group 2, the FLAG-NRF2-induced mRNA expressions of these genes were further induced by co-expression of HER2CA (Figure 2E); 5) the mRNA expression of MRP4 in group 5 was not induced by either FLAG-NRF2 or HER2CA alone. However, the MRP4 mRNA expression was weakly but significantly induced by co-expression of FLAG-NRF2 and HER2CA (Figure 2F). These results suggest that genes having roles in drug resistance are regulated by differential cooperation of NRF2 and HER2 (Figure 2G). The expressions of genes in group 3 may be regulated by either NRF2 or HER2CA, independently. However, the expressions of these genes can be further regulated by NRF2 and HER2CA in a cooperative manner. Importantly, the regulation of mRNA expression of genes in groups 4 and 5 can be achieved by another level of regulation. Although HER2CA alone does not affect the expression of group 4 genes, it can increase the NRF2-dependent transcription of these genes either by signal crosstalk or by direct interaction.

### HER2CA binds to NRF2

Since it has been known that NRF2 stability/activity is regulated by various protein-protein interactions^17^,^18^,^19^,^20^,^21^,^22^, we tested whether HER2CA interacts with NRF2. HEK293T cells were transiently transfected with FLAG-NRF2 and HER2CA expression vectors and subjected to immunoprecipitation. Unexpectedly, co-expression of HER2CA with FLAG-NRF2 was increased the level of FLAG-NRF2 in HEK293T cells compared to expression of FLAG-NRF2 without HER2CA (Figure 3A, upper panel). Under these conditions, immunoprecipitation of HER2CA revealed that HER2CA binds to FLAG-NRF2 (Figure 3A lower panel). The increased expression of FLAG-NRF2, observed in HEK293T cells transfected with FLAG-NRF2 and HER2CA expression vectors, might be due to increased stability of FLAG-NRF2 protein. Similarly, increase of both endogenous and exogenous NRF2 protein by HER2CA expression was observed in MCF7 cells (Figure 1A).

To reduce proteasomal degradation of NRF2 protein, we used an alternative fusion-tag for NRF2 expression. Since the green fluorescent protein (GFP), as a fusion tag, has been known to inhibit polyubiquitination and subsequence proteasomal degradation of fusion proteins^34^, we further determine the HER2CA-NRF2 binding with overexpression of GFP-NRF2. As expected, higher level of NRF2 protein was detected when cells were transfected with GFP-NRF2 expression vector (Figure 3B lane 3, upper panel) than when cells were transfected with FLAG-NRF2 expression vector (Figure 3A lane 3, upper panel). Again, immunoprecipitation of GFP proteins demonstrated that GFP-NRF2 physically interacts with HER2CA in these cells (Figure 3B, lower panel). Consistently, reciprocal immunoprecipitation of HER2CA also revealed that GFP-NRF2 binds to HER2CA (data not shown).

The interaction of endogenous HER2 and NRF2 was further determined in two HER2-amplified breast cancer cells. AU-565 and SK-BR-3 cells were cultured in serum-starved condition for 24 hr and stimulated by 100 ng/ml of heregulin (HRG) for 4 hr. And the cell lysates were subjected to immunoprecipitation of HER2 and NRF2, respectively. The activation of HER2 was monitored by detecting phospho-AKT (S473) (Figure 3C). Under these conditions, HER2 bound to NRF2 even in the absence of HRG stimulation. Moreover, an increased HER2-NRF2 binding was observed in the presence of HRG. These results suggest that HER2 physically interacts with NRF2 in HER2-positive breast cancer cells.

### HER2CA confers drug resistance of MCF7 cells in an NRF2-dependent manner

**Figure 4:**
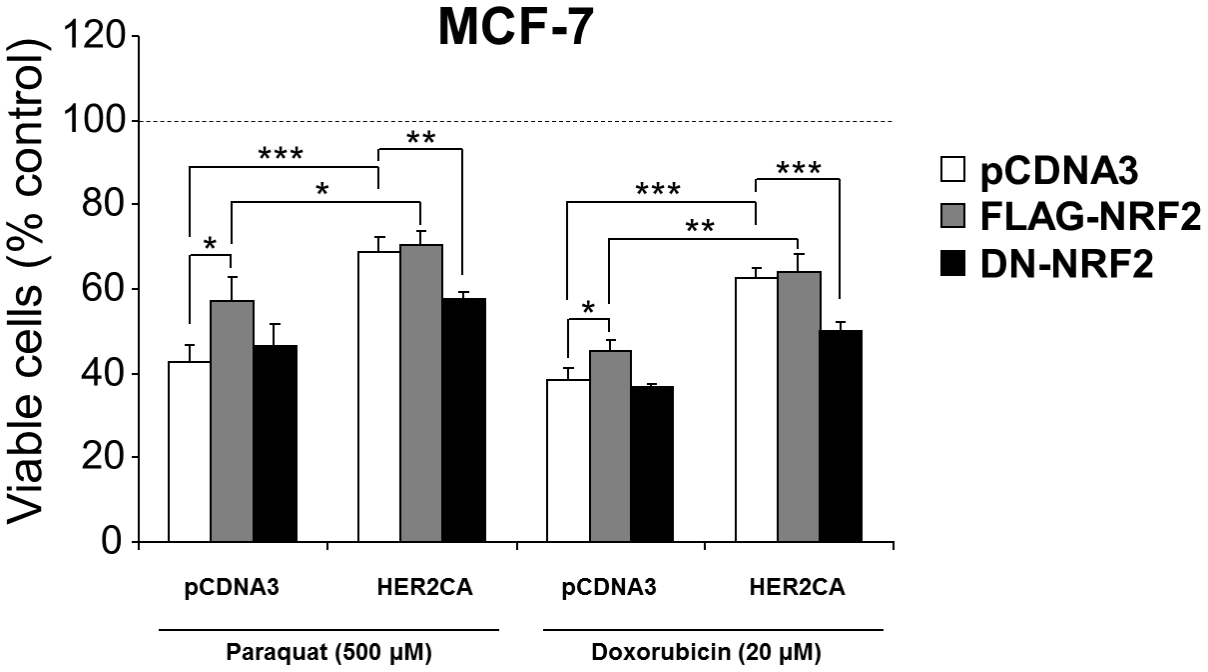
HER2CA confers drug-resistance of MCF7 cells in an NRF2-depenent manner. MCF7 cells were transfected with expression vectors as indicated and incubated with increasing concentrations of drugs for 48 hr. Cell viability was determined by MTT assay. Representative data are shown as mean ± SEM of triplicate experiments. Statistically significance was determined by two-tailed Student's t-test. *P ≤ 0.05; **P ≤ 0.01; and ***P ≤ 0.001.

Since both HER2 and NRF2 give drug resistances of cancer cells, respectively, we determined the drug sensitivity of MCF7 cells by MTT assay, after transient expression of HER2CA or FLAG-NRF2 in the presence of an oxidative stress-inducing agent paraquat or a chemotherapeutic agent doxorubicin. As results, overexpression of FLAG-NRF2 marginally but significantly induced the resistance of MCF7 cells to both paraquat and doxorubicin (Figure 4). Although the transfection increased the basal drug resistance of MCF7 cell lines, the results were obtained from repeated experiment and statistically significant. Overexpression of HER2CA markedly induced the resistance of MCF7 cells to these drugs. However, this induced resistance of MCF7 cells was saturated by HER2CA and no additional increase was observed when HER2CA and FLAG-NRF2 were co-expressed.

Since overexpression of HER2CA itself saturated drug resistance of MCF7 cells, drug sensitivity was further determined in MCF7 cells transiently transfected with HER2CA expression vector in the absence or presence of DN-NRF2 expression. Although the expression of DN-NRF2 alone had little or no effect on the basal drug sensitivity of MCF7 cells, co-expression of DN-NRF2 antagonized the HER2CA-induced drug resistance of MCF7 cells to both paraquat and doxorubicin (Figure 4). These results suggest that active HER2 (HER2CA) contribute to resistance of MCF7 cells to these drugs in an NRF2-dependent manner.

Taken together, these results suggest that active HER2 (HER2CA) binds to NRF2 to activate the transcription of a set of genes which are involved in drug resistance of cancer cells at multiple levels. Various studies have demonstrated that HER2 or NRF2, independently, gives resistance of cancer cells to a range of therapeutics. However, to the best of our knowledge no direct link, between HER2 and NRF2 in drug resistance of cancer cells, has ever been reported. In the present study, we first demonstrated the direct link between HER2 and NRF2 in drug resistance of cancer cells. Our present study suggests that HER2 may regulate NRF2 stability and/or activity through direct physical interaction. This HER2-NRF2 interaction may contribute to 1) stabilization of NRF2 by inhibiting or competing with KEAP1 similar to other proteins as previously reported^17^,^18^,^19^,^20^,^21^,^22^. Data presented in this study support this possibility: 1) the expression of FLAG-NRF2 protein was increased in HER2CA-transfected MCF7 and HEK293T cells and a GFP-tag, which inhibits polyubiquitination-dependent proteasomal degradation, increased NRF2 protein expression in HEK293T cells even in the absence of HER2CA expression; 2) enhancing translocation of NRF2 into nucleus; or 3) recruitment of HER2, as a coactivator, to the promoters of NRF2-target genes. In fact, it has been reported that HER2 is localized in the nucleus of cells to act as a transcriptional activator^35^,^36^,^37^,^38^,^39^. Further studies are needed to determine the exact roles of HER2-NRF2 interaction in regulation of drug resistance in HER2-amplified or -activated human cancer cells.

## Methods

### Cell lines and reagents

Cell culture reagents were purchased from Invitrogen (Carlsbad, CA) or Lonza (Basel, Switzerland). Cell lines were obtained from the Tissue Culture Shared Resource at Georgetown University Medical Center. MCF7 and HEK293T cells were maintained in DMEM containing 5% heat inactivated fetal bovine serum (HI-FBS; from HyClone, Logan, UT or Omega Scientific, Tarzana, CA), 100 units/ml penicillin and 100 µg/ml streptomycin. AU-565 cells were maintained in RPMI1640 containing 5% HI-FBS, 100 units/ml penicillin and 100 µg/ml streptomycin. SK-BR-3 cells were maintained in McCoy's 5A containing 10% HI-FBS, 100 units/ml penicillin and 100 µg/ml streptomycin. Cell viability was monitored by Luna Automated Cell Counter (Logos Biosystems, Gyunggi-Do, Korea). Heregulin 1-β1 (HRG) was purchased from R&D Systems (Minneapollis, MN) and reconstituted as recommendation. tert-bytylhydroquinone (tBHQ) and paraquat were obtained from Sigma (St. Louis, MO) and dissolved in dimethyl sulfoxide (DMSO). Doxorubicin was purchased from Sigma and dissolved in distilled water.

### Plasmid DNAs, transfection and reporter gene assay

Expression vector for HER2CA (V659E)^30^ was obtained from Addgene (Cambridge, MA). Plasmid DNAs for FLAG-NRF2, GST-NRF2, DN-NRF2, ARE-Luc, and HO-1 promoter-Luc were described elsewhere^16^,^31^,^32^. For reporter gene assay, DNAs were transfected with Lipofectamine Plus reagent (Invitrogen). Luciferase activity was determined as recommended by manufacturer (Promega, Madison, WI) using Victor2 plate reader (Perkin-Elmer, Waltham, MA) at the Genomics and Epigenomics Shared Resource of Georgetown University Medical Center and normalized to protein concentrations.

### Western blots, antibodies and immunoprecipitation

Western blot analyses were performed using 25 ~ 50 μg of cleared cell lysates as described previously^28^. Antibodies used in this study were purchased from the following sources: phospho-AKT (S473) (#9271), AKT (#9272), phospho-ERK1/2 (T202/Y204) (#4370), and HER2 (#2247) from Cell Signaling Technologies, Inc. (Danvers, MA); HO-1 (ADI-OSA-110) from Enzo Life Sciences (Farmingdale, NY); β-actin, ERK1 (sc-94), NRF2 (sc-13032), and MRP5 (sc-5770) from Santa Cruz Biotechnology (Santa Cruz, CA); FLAG M2 (F1804) from Sigma; and GFP (ab290-50) from Abcam (Cambridge, MA). Chemiluminescence reagent was purchased from Santa Cruz Biotechnology or Thermo Scientific (Rockford, IL). Immunoprecipitation was performed as described previously^16^.

### DNA binding assay

NRF2 DNA-binding assay was performed by TransAM NRF2 assay Kit (Active Motif, Carlsbad, CA) according to the manufacturer's manual.

### siRNA transfection

Transfection of siRNA was performed with Lipofectamine 2000 (Invitrogen) as described previously^25^. After 3-day incubation, the transfected cells were re-seeded and transfected with plasmid DNAs by Lipofectamine 2000 as indicated. To maintain KEAP1 knockdown, freshly prepared siRNA transfection was added to the cells. The control- and KEAP1-siRNA were obtained from Dharmacon, Inc (Lafayette, CO). Sequences of siRNAs were as follows; control-siRNA 5′–GAC GAG CGG CAC GUG CAC A–3′, and KEAP1-siRNA 5′– GGG AGU ACA UCU ACA UGC A–3′.

### 3-(4,5-Dimethylthiazol-2-yl)-2,5-diphenyltetrazolium bromide (MTT) assays

After transfection of DNAs, cells were further treated with indicated concentrations of drug for 48 hr. Cell proliferation was determined by MTT assay as described previously^24^. The absorbance of each well was measured by ELx808 Microplate Reader (BioTek, Winooski, VT). Viable cells were calculated as percent of control, vehicle-treated cells.

### Reverse-transcription quantitative polymerase chain reaction (RT-qPCR)

RT-qPCR was performed in triplicate with the Fast SYBR green master mix (Applied Biosystems, Carlsbad) using an Applied Biosystems-Prism Sequence Detector System 7700 at the Genomics and Epigenomics Shared Resource of Georgetown University Medical Center and analyzed with SDS software as previously described^28^. The following primers were used: HER2 forward, 5′-acc ggc aca gac atg aag ct-3′ and reverse, 5′-agg aag gac agg ctg gca tt-3′; NRF2 forward, 5′-aaa cca ccc tga aac gac ag-3′ and reverse, 5′-agc ggc ttg aat gtt tgt c-3′; HO-1 forward, 5′-agg tca tcc cct aca cac ca-3′ and reverse, 5′-tgt tgg gga agg tga aga ag-3′; NQO1 forward, 5′-gca ctg atc gta ctg gct ca-3′ and reverse, 5′-tga aca ctc gct caa acc ag-3′; GCLC forward, 5′-ctg ggg agt gat ttc tgc at-3′ and reverse, 5′-agg agg ggg ctt aaa tct ca-3′; MRP1 forward, 5′-acc aag acg tat cag gtg gcc-3′ and reverse, 5′-ctg tct ggg cat cca gga t-3′; MRP2 forward, 5′-tga aag gct aca agc gtc ct-3′′ and reverse, 5′-gag ccg cag tga ata aga gg-3′; MRP4 forward, 5′-gag gga tga att tgg ctt ca-3′ and reverse, 5′-cag ggc tgc tga gac aca ta-3′; MRP5 forward, 5′-acc cgt tgt tgc cat ctt ag-3′ and reverse, 5′-tct gtc aac agc cac tga gg-3′; MDR1 forward, 5′-tga cat tta ttc aaa gtt aaa agc a-3′ and reverse, 5′-tag aca ctt tat gca aac att tca a-3′; GSTA2 forward, 5′-ggc tgc agc tgg agt aga gt-3′ and reverse, 5′-aag gca ggg aag tag cga tt-3′; CYP3A4 forward, 5′-caa gac ccc ttt gtg gaa aa-3′ and reverse, 5′-cga ggc gac ttt ctt tca tc-3′; GSTP1 forward, 5′-cac caa cta tga ggc ggg caa-3′ and reverse, 5′-atc agc agc aag tcc agc a -3′; and GAPDH forward, 5′-gta tga caa cga att tgg cta cag -3′ and reverse, 5′-agc aca ggg tac ttt att gat ggt-3′. The GAPDH primer was used as an internal control.

### Statistical analysis

Two-tailed Student's t-test was applied for statistical analysis. *indicates *P* < 0.05; **indicates *P* < 0.01; and *** indicates *P* < 0.001.

## Author Contributions

The late I.B. who was the initial corresponding author sadly deceased after the submission of this manuscript, he awarded the research grant initiating this experiment and supervised it. H.J. Kang Y.W.Y. and Y.J.J. designed experiments; Y.W.Y., H.J.Kang,Y.B.H. and H.J.Kim performed the experiments and analyzed data. Y.W.Y., Y.S.S., I.B. wrote manuscript. All authors discussed the results and reviewed the manuscript.

## Supplementary Material

Supplementary Information supplementary information
