# Supplementary material for: HER2 confers drug resistance of human breast cancer cells through activation of NRF2 by direct interaction
Source: Sci Rep. 2014 Dec 3;4:7201. doi: 10.1038/srep07201 (PMC4252900; doi:10.1038/srep07201)
Supplement: Supplementary Information [file srep07201-s1.pdf]

HER2 confers drug resistance of human breast cancer cells through activation of NRF2 by direct interaction

Hyo Jin Kang[1,**], Yong Weon Yi[1,3,**], Young Bin Hong[1,4], Hee Jeong Kim[1], Young-Joo Jang[3], Yeon-Sun Seong[1,3], and Insoo Bae[1,2,3,*]

[1]Department of Oncology and [2]Department of Radiation Medicine, Lombardi Comprehensive Cancer Center, Georgetown University, Washington DC, 20057, USA.
[3]Department of Nanobiomedical Science and BK21 PLUS Research Center for Regenerative Medicine, Dankook University, Cheonan, Korea.
[4]Present address: Department of Neurology, Sungkyunkwan University School of Medicine, Seoul, Korea.

[**]These authors contributed equally to this work.

*Correspondence to: Insoo Bae, Associate Professor in the Department of Oncology, Lombardi Comprehensive Cancer Center, Georgetown University Medical Center, Washington, DC 20057, USA, Tel.: +1 202 687 5267, Fax: +1 202 687 2847, E-mail: ib42@georgetown.edu

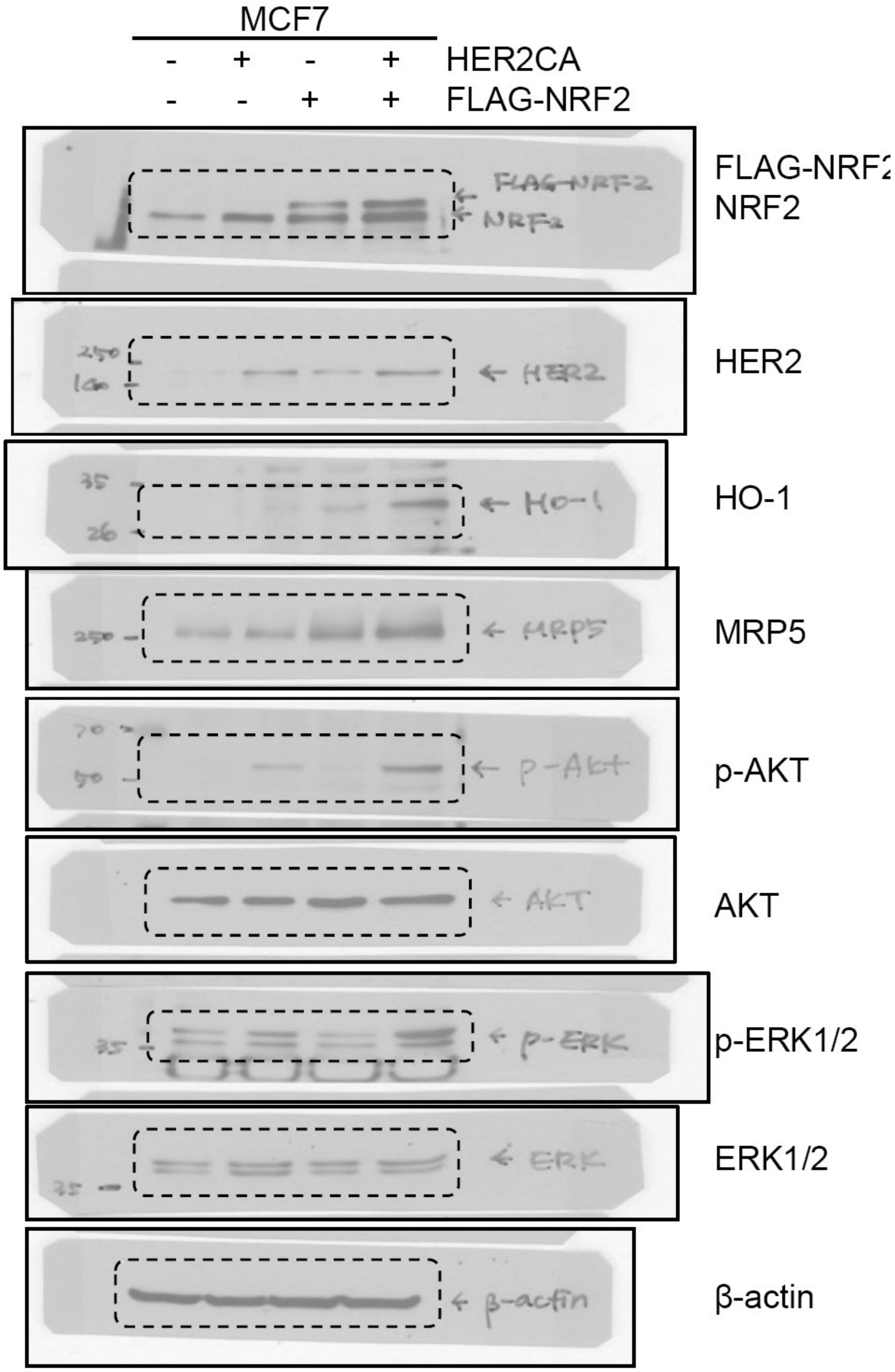

Supplementary Figure S1 Full size images of the cropped blots presented in figure 1A. The excised blots (demarcated by linear lines) after electrotransfer were probed with indicated antibodies and the images in dashed lines were presented figure 1A

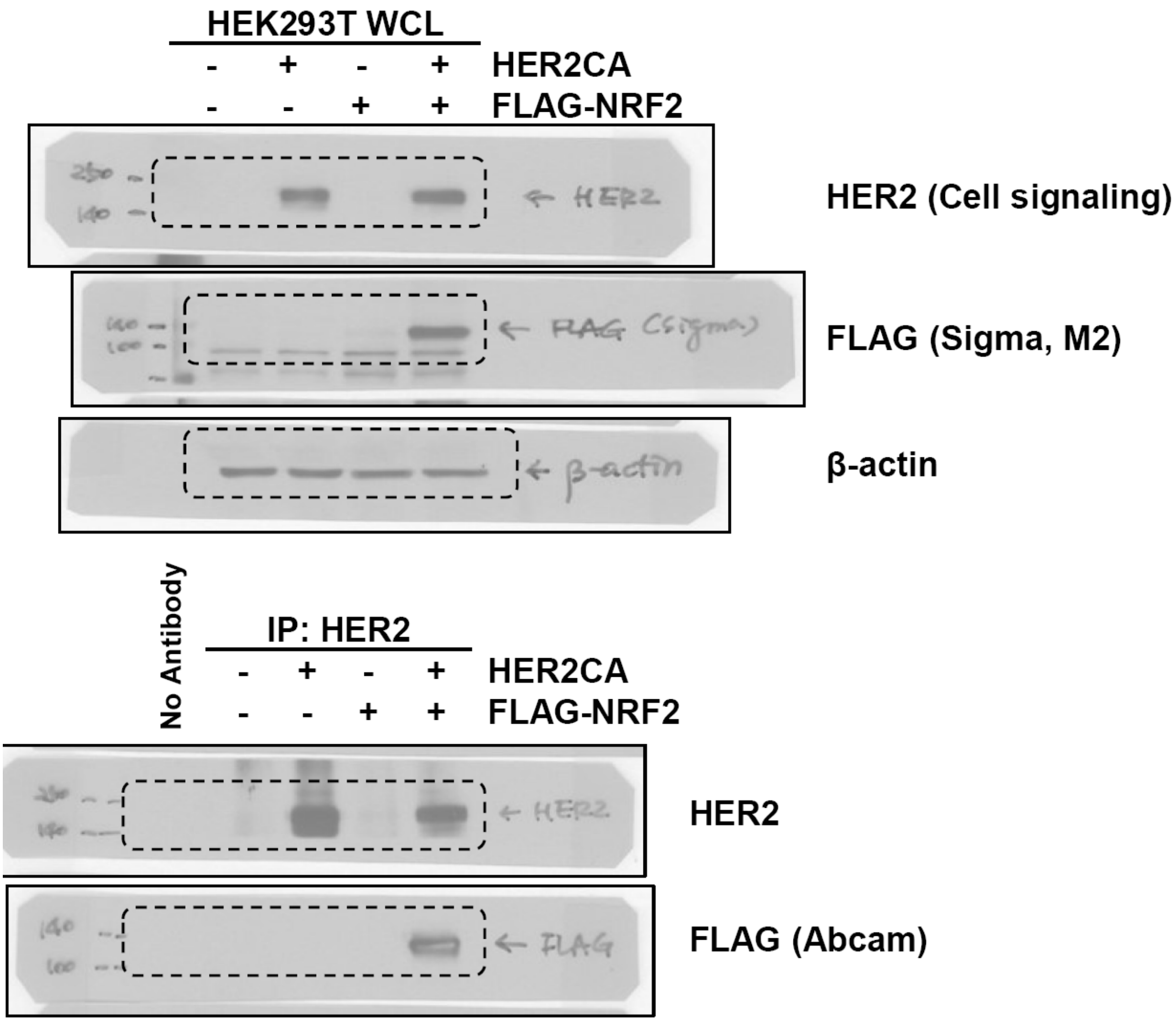

Supplementary Figure S2 Full size images of the relevant cropped blots presented in figure 3A. The excised blots (demarcated by unbroken lines) after electrotransfer were probed with indicated antibodies and the representative images in dashed lines were presented figure 3A, The FLAG immune-blot (FLAG (Abcam)) image was replaced with same blots with different time of exposure.

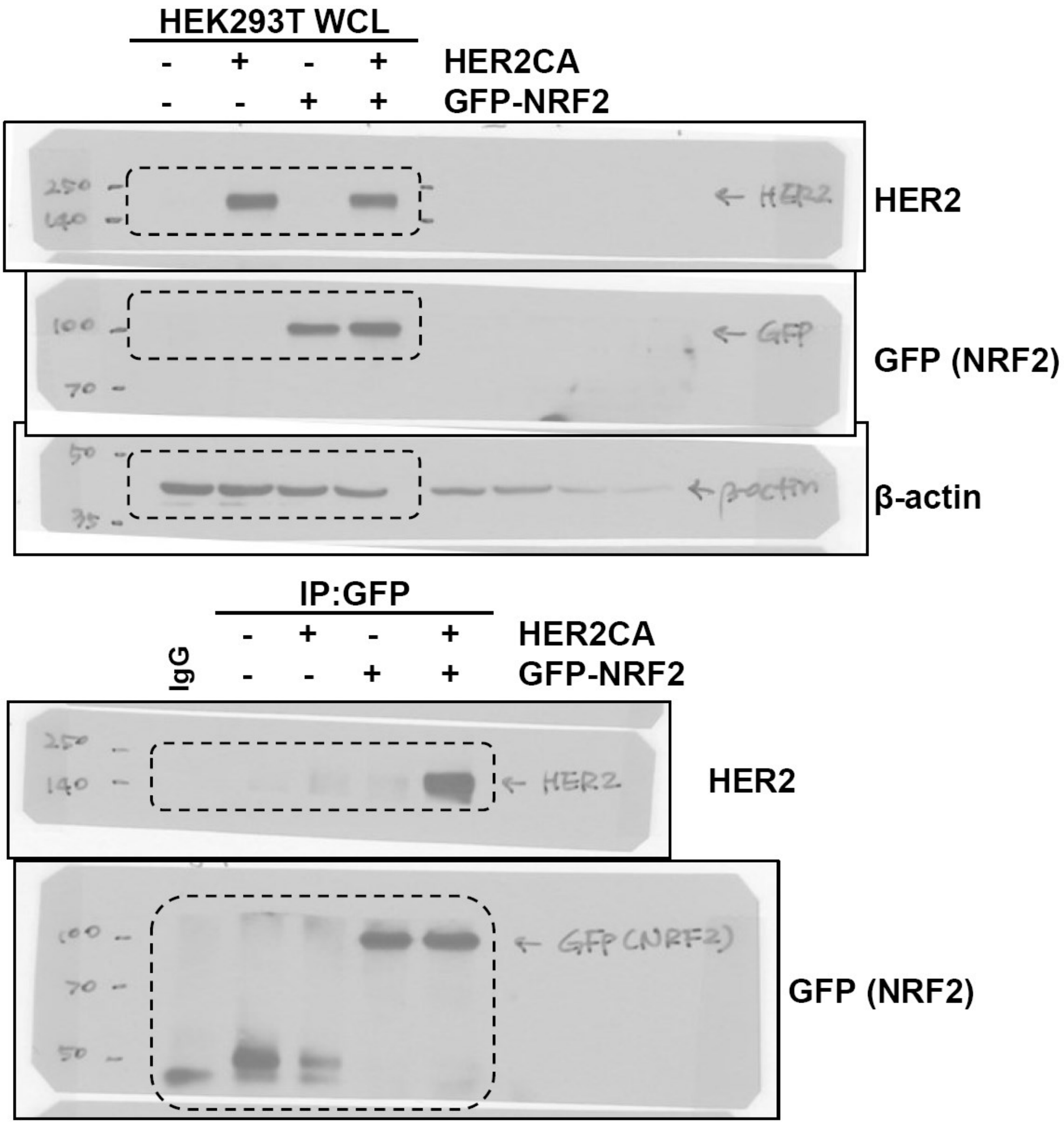

Supplementary Figure S3 Full size images of the cropped blots presented in figure 3B. The excised blots (demarcated by linear line) after electrotransfer were probed with indicated antibodies and the images in dashed lines were presented figure 3B

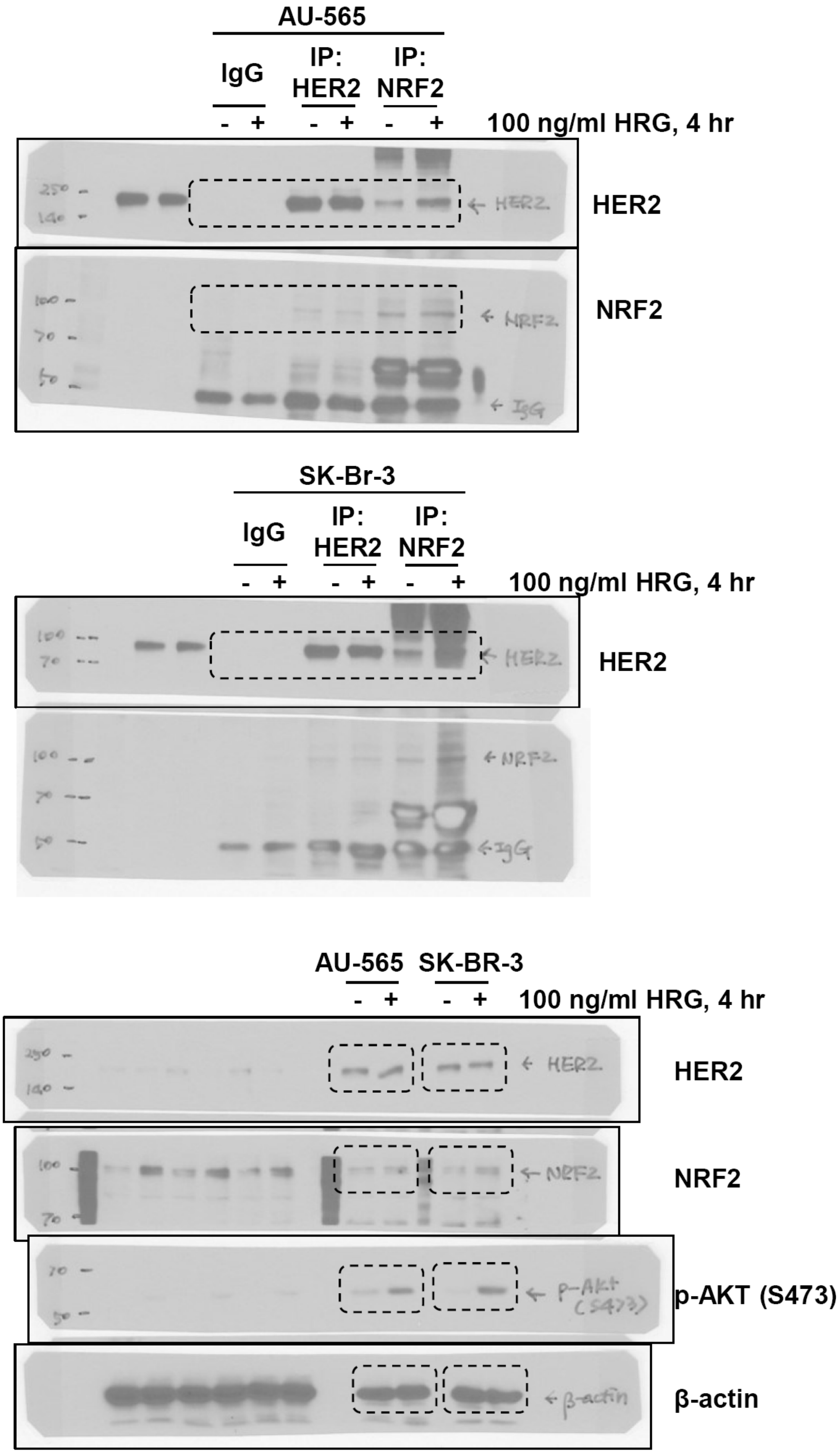

Supplementary Figure S4 Full size images of the cropped blots presented in figure 3C. The excised blots (demarcated by unbroken line) after electrotransfer were probed with indicated antibodies and the images in dashed lines were presented figure 3C
